# Supplementary material for: Acetylation discriminates disease-specific tau deposition
Source: Nat Commun. 2023 Sep 22;14:5919. doi: 10.1038/s41467-023-41672-1 (PMC10517010; doi:10.1038/s41467-023-41672-1)
Supplement: Supplementary file 1 — Supplementary Information [file 41467_2023_41672_MOESM1_ESM.pdf]

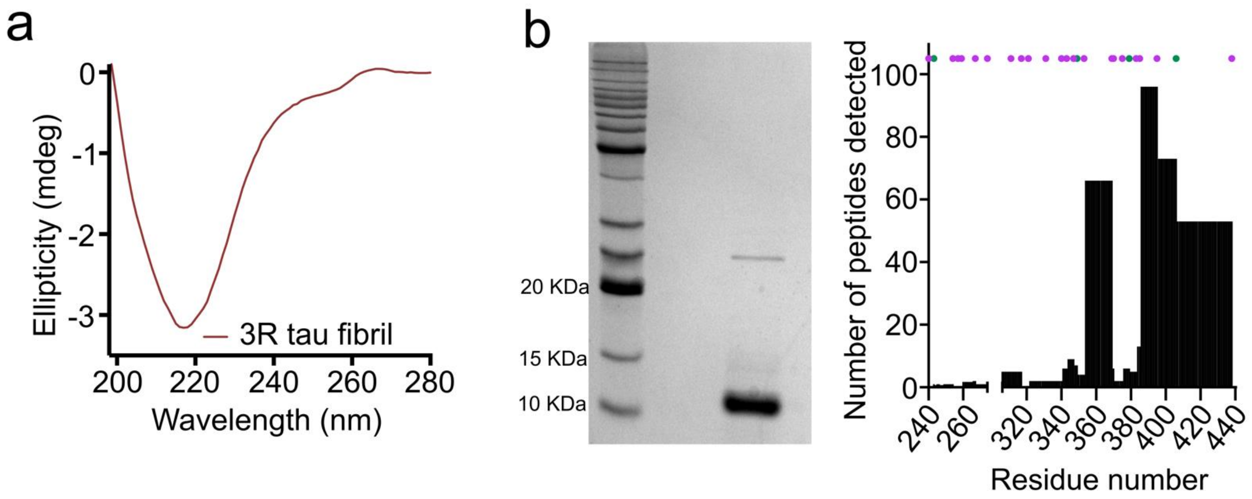

Supplementary Fig. 1 │ CD spectra and trypsin digestion of 3R tau fibrils. a, CD spectra of 3R tau fibrils. Source data are provided as a Source Data file. **b**, SDS-PAGE gel of trypsin digested 3R tau fibrils. The protease digestion experiment has been performed up to 3 times with similar results. Numbers of detected peptides are shown to the right. The position of lysine and arginine residues are marked with purple and green dots, respectively. Source data are provided as a Source Data file.

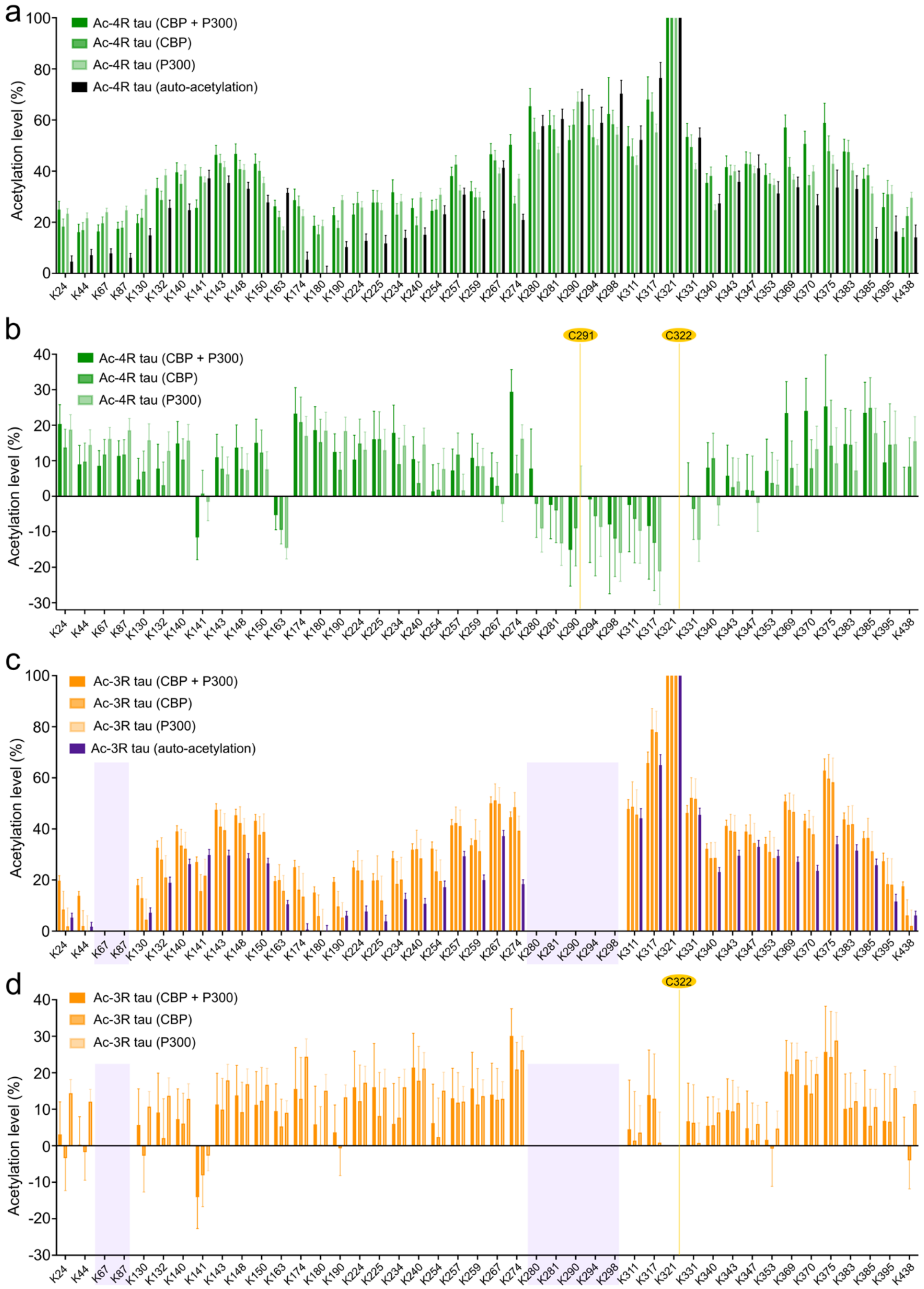

Supplementary Fig. 2 │ Acetylation levels of 4R and 3R tau. a, Analysis of acetylation levels of individual lysine residues present in 4R tau upon acetylation with either p300, or CBP acetyltransferases, or both, or in the absence of any acetyltransferases (auto-acetylation). The acetylation levels of individual lysine residues were determined from the intensity ratio of the unacetylated lysine (in the acetylated sample) and unmodified lysine. The error of the intensity ratio for each lysine residue was calculated from the signal-to-noise ratio of the cross-peaks in the spectra. Source data are provided as a Source Data file. **b**, Difference between the acetylation levels of 4R tau when acetylated in the presence or absence of acetyltransferases. The difference was calculated by subtracting the acetylation level of individual lysine residues in the absence of acetyltransferases (i.e.,

in the condition of autoacetylation) from the acetylation level in the presence of acetyltransferases. Source data are provided as a Source Data file. **c**, Analysis of acetylation levels of individual lysine residues present in 3R tau upon acetylation with either p300, or CBP acetyltransferases, or both, or in the absence of any acetyltransferases (autoacetylation). The acetylation level of individual lysine residues was determined from the intensity ratio of the unacetylated lysine (in the acetylated sample) and unmodified lysine. The error of the intensity ratio for each lysine residue was calculated from the signal-to-noise ratio of the cross-peaks in the spectra. The lysine residues not present in the 3R tau but present in the 4R tau are highlighted with purple boxes. Source data are provided as a Source Data file. **d**, Difference between the acetylation levels of 3R tau when acetylated in the presence or absence of acetyltransferases. The difference was calculated by subtracting the acetylation level of individual lysine residues in the absence of acetyltransferases (i.e., in the condition of autoacetylation) from the acetylation level in the presence of acetyltransferases. Lysine residues not present in the 3R tau but present in the 4R tau are highlighted with purple boxes. Source data are provided as a Source Data file.

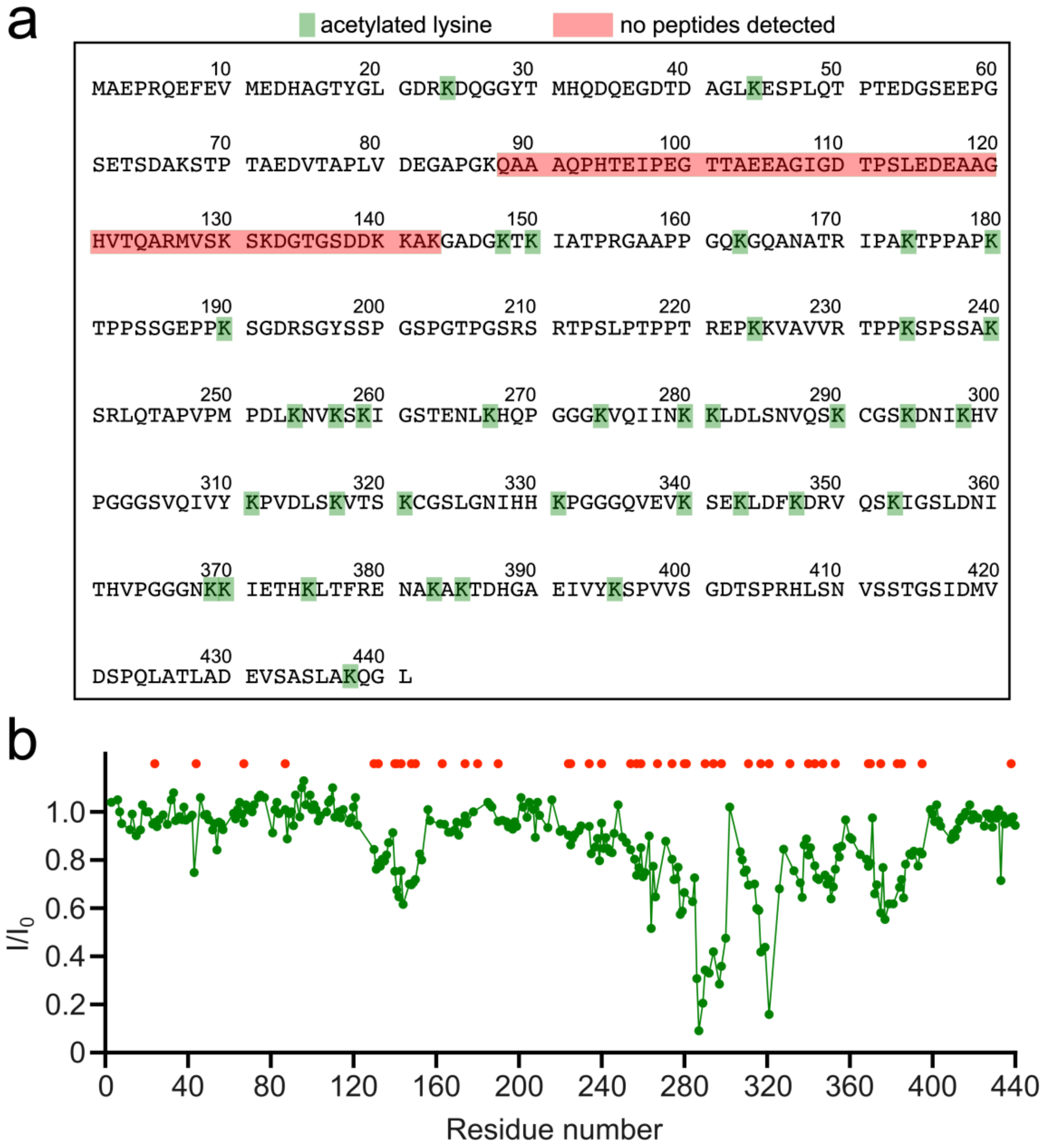

Supplementary Fig. 3 │ Acetylation of 4R tau. a, Detection of the acetylation sites in 4R tau by mass spectrometry upon acetylation with both p300 and CBP acetyltransferases for twelve hours. The acetylated 4R tau sample was digested with trypsin enzyme and the digested peptides were detected by ESI-MS. The acetylated lysine residues are highlighted with green colour. The region for which no peptides are detected is highlighted with red colour. A detailed list of the detected peptides is available in Supplementary Data 1. **b**, Residue specific intensity ratio plot of acetylated -^{15}N labeled-4R tau. The intensity ratio is calculated by dividing the cross-peak intensities observed in the HSQC spectrum of the acetylated 4R tau by the unmodified 4R tau. The acetylation reaction of the ^{15}N-labeled 4R tau was performed in the presence of both p300 and CBP acetyltransferases for twelve hours. The location of the lysine residues present in the sequence of 4R tau is indicated with red dots. Source data are provided as a Source Data file.

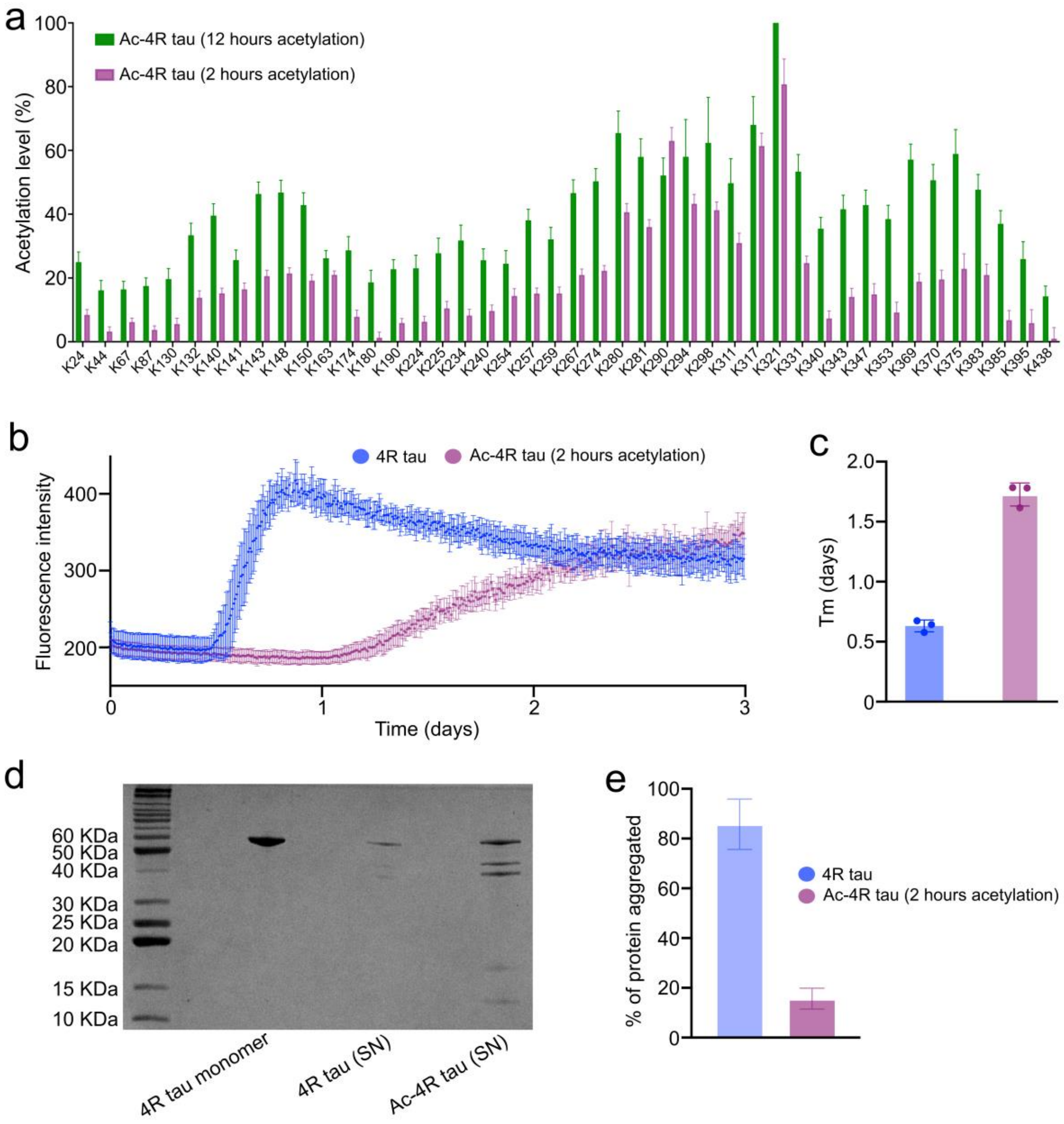

Supplementary Fig. 4 | Acetylation of the most reactive lysine residues of 4R tau. a, Analysis of acetylation levels of individual lysine residues present in 4R tau upon acetylation with both p300 and CBP acetyltransferases for 2 hours (magenta) or 12 hours (green). The acetylation levels of individual lysine residues were determined from the intensity ratio of the unacetylated lysine (in the acetylated sample) and unmodified lysine. The error of the intensity ratio for each lysine residue was calculated from the signal-to-noise ratio of the cross-peaks in the spectra. Source data are provided as a Source Data file. **b**, Fibrillization kinetics of unmodified 4R tau (blue) and 2-hour-acetylated 4R tau (magenta) followed by ThT fluorescence. Acetylation reactions were performed in the presence of p300/CBP. Protein concentrations were 25 µM. Error bars represent the std of three independent samples. The centre of the error bars represents the average value of n=3 independent samples. The aggregation assay has been repeated up to 2 times with similar results. Source data are provided as a Source Data file. **c**, Aggregation half-time (Tm) to reach steady-state ThT fluorescence intensity of unmodified 4R tau (blue) and 2-hour-acetylated 4R tau (magenta). The aggregation assay has been repeated up to 2 times with similar results. Data are presented as mean +/- std of n=3 independent samples. Source data are provided as a Source Data file. **d**, SDS PAGE gel of monomer and supernatant (SN) (after pelleting down the fibrils) of unmodified and 2-hour-acetylated 4R tau. The fibril samples were taken at the end of aggregation. The experiment has been repeated one time with similar results. Source data are provided as a Source Data file. **e**, Amount of protein aggregated. Error bars were calculated from the signal-to-noise ratio of the bands in the SDS-PAGE gel shown in (d). Source data are provided as a Source Data file.

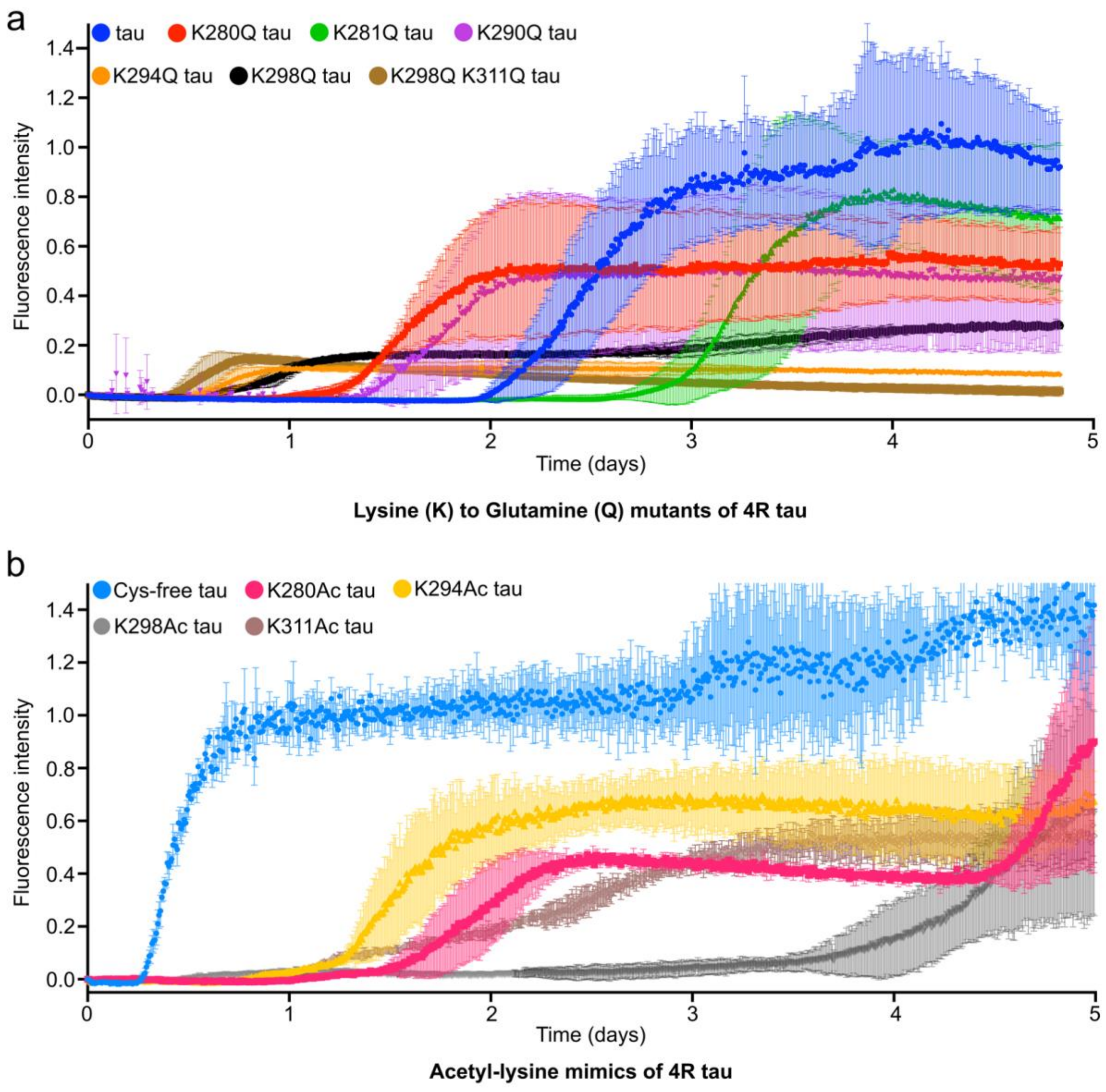

Supplementary Fig. 5 │ Aggregation of lysine (K) to glutamine mutants (Q) and acetyl-lysine mimics of tau. a, Aggregation kinetics of 25 µM unmodified and different K-to-Q mutants of 4R tau. Error bars represent the std of three independently aggregated samples. The centre of the error bars represents the average value of n=3 independent samples. Source data are provided as a Source Data file. **b**, Aggregation kinetics of 25 µM C291S/C322S- (Cys-free) tau and several single-site acetyl-lysine mimics of tau. Error bars represent the std of three independently aggregated samples. The centre of the error bars represents the average value of n=3 independent samples. Source data are provided as a Source Data file.

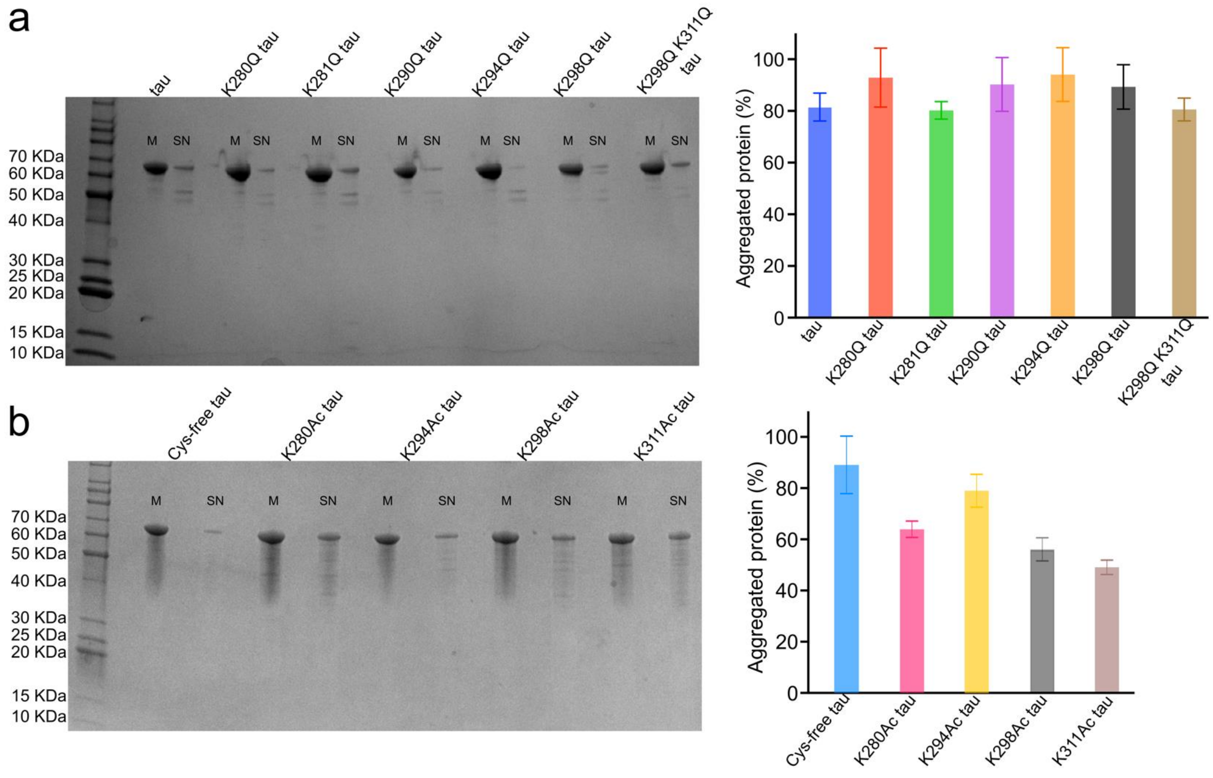

Supplementary Fig. 6 | SDS-PAGE analysis to determine the amount of aggregated protein. a, (left) SDS PAGE gel of monomer (M) and supernatant (SN) (after pelleting down the fibrils) of unmodified and different K-to-Q mutants of tau. The fibril samples were taken after five days of aggregation. (right) Amount of protein aggregated. Error bars were calculated from the signal-to-noise ratio of the bands. The experiment has been repeated one time with similar results. Source data are provided as a Source Data file. **b**, (left) SDS PAGE gel of monomer (M) and supernatant (SN) (after pelleting down the fibrils) of C291S/C322S- (Cys-free) tau and several single-site acetyl-lysine mimics of tau. Fibril samples were taken after five days of aggregation. (right) Amount of protein aggregated. Error bars were calculated from the signal-to-noise ratio of the bands. The experiment has been repeated one time with similar results. Source data are provided as a Source Data file.

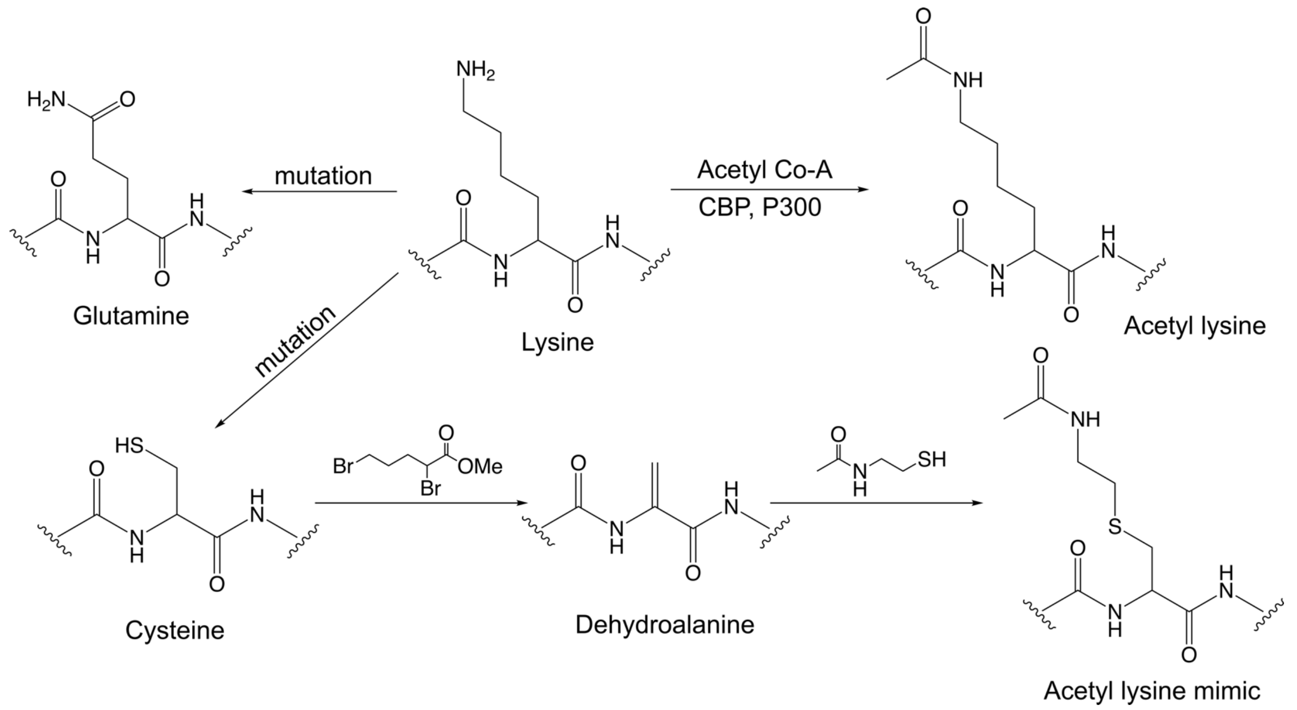

Supplementary Fig. 7 | Reaction scheme to prepare acetyl-lysine mimics. The traditional approach of mimicking lysine acetylation by mutating lysine residue to glutamine is also shown.

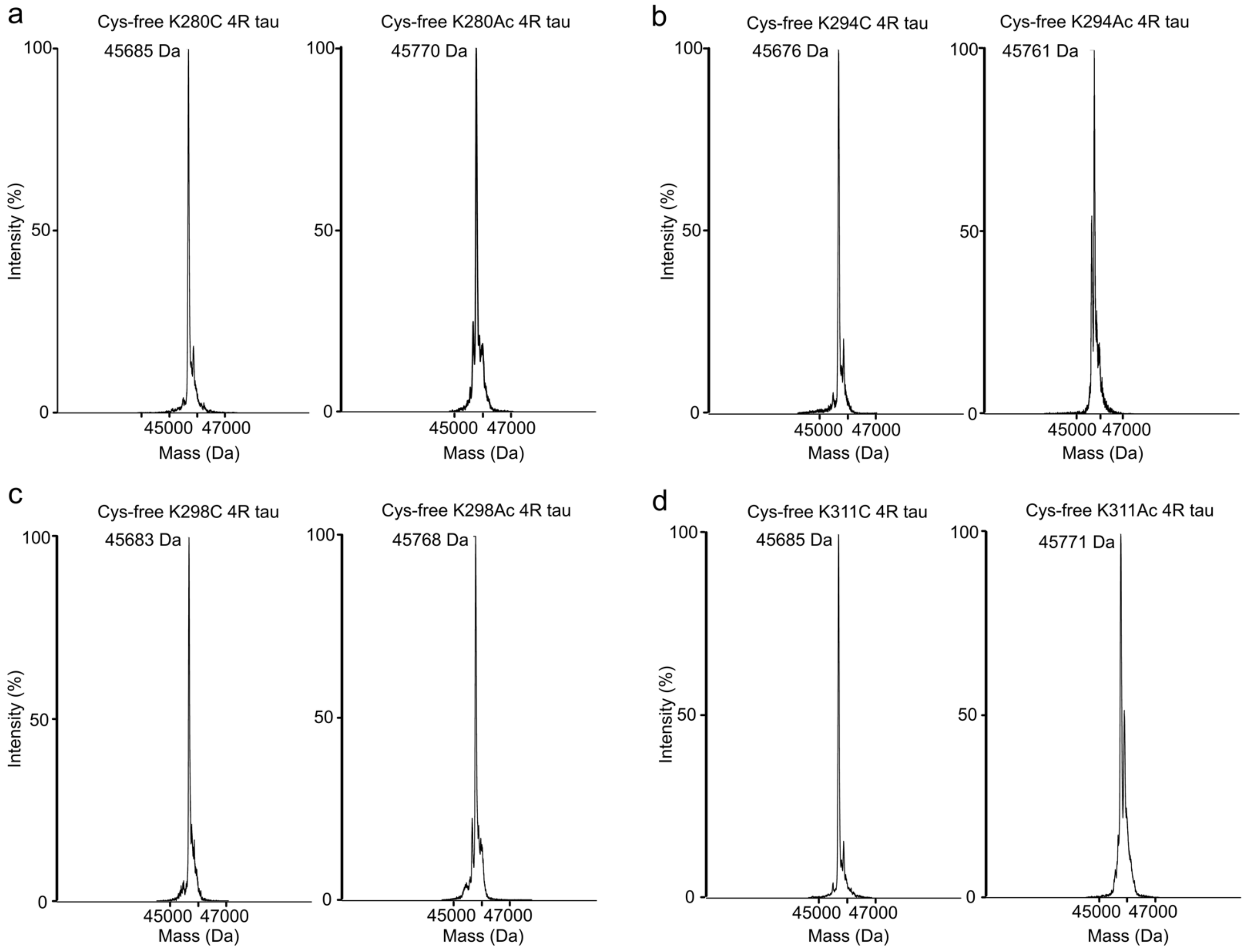

Supplementary Fig. 8 | Mass spectra of acetyl-lysine mimics of 2N4R tau. a, Mass spectra of Cys-free K280C 4R tau before and after acetyl-lysine mimic formation. **b**, Mass spectra of Cys-free K294C 4R tau before and after acetyl-lysine mimic formation. **c**, Mass spectra of Cys-free K298C 4R tau before and after acetyl-lysine mimic formation. **d**, Mass spectra of Cys-free K311C 4R tau before and after acetyl-lysine mimic formation.

Mutant	Forward primer	Reverse Primer	Template
K280Q 2N4R tau	5' GGTGCAGATAATTAATCAGAAGCTGGATCTTAGC 3'	5' GCTAAGATCCAGCTTCTGATTAATTATCTGCACC 3'	2N4R tau
K281Q 2N4R tau	5' GTGCAGATAATTAATAAGCAGCTGGATCTTAGCAACG 3'	5' CGTTGCTAAGATCCAGCTGCTTATTAATTATCTGCAC 3'	2N4R tau
K290Q 2N4R tau	5' CAACGTCCAGTCCCAGTGTGGCTCAAAG 3'	5' CTTTGAGCCACACTGGGACTGGACGTTG 3'	2N4R tau
K294Q 2N4R tau	5' GTCCAAGTGTGGCTCACAGGATAATATCAAAC 3'	5' GTTTGATATTATCCTGTGAGCCACACTTGGAC 3'	2N4R tau
K298Q 2N4R tau	5' CAAAGGATAATATCCAACACGTCCCGGG 3'	5' CCCGGGACGTGTTGGATATTATCCTTTG 3'	2N4R tau
K298Q K311Q 2N4R tau	5' GTGCAAATAGTCTACCAACCAGTTGACCTG 3'	5' CAGGTCAACTGGTTGGTAGACTATTTGCAC 3'	K298Q 2N4R tau
C291S 2N4R tau	5' CAACGTCCAGTCCAAGTCAGGCTCAAAGGATAATATC 3'	5' GATATTATCCTTTGAGCCTGACTTGGACTGGACGTTG 3'	2N4R tau
C291S C322S 2N4R tau	5' GTGACCTCCAAGTCTGGCTCATTAGGC 3'	5' GCCTAATGAGCCAGACTTGGAGGTCAC 3'	C291S 2N4R tau
C291S C322S K280C 2N4R tau	5' GGTGCAGATAATTAATTGCAAGCTGGATCTTAGC 3'	5' GCTAAGATCCAGCTTGCAATTAATTATCTGCACC 3'	C291S C322S 2N4R tau
C291S C322S K294C 2N4R tau	5' CCAGTCCAAGTCAGGCTCATGTGATAATATCAAACACGTC 3'	5' GACGTGTTTGATATTATCACATGAGCCTGACTTGGACTGG 3'	C291S C322S 2N4R tau
C291S C322S K298C 2N4R tau	5' CAAAGGATAATATCTGTCACGTCCCGGGAGGC 3'	5' GCCTCCCGGGACGTGACAGATATTATCCTTTG 3'	C291S C322S 2N4R tau
C291S C322S K311C 2N4R tau	5' GTGTGCAAATAGTCTACTGTCCAGTTGACCTGAGCAAG 3'	5' CTTGCTCAGGTCAACTGGACAGTAGACTATTTGCACAC 3'	C291S C322S 2N4R tau

Supplementary Table. 1 │ Details of the primers used to generate different mutants of tau.
