# Supplementary material for: Acetylation discriminates disease-specific tau deposition
Source: Nat Commun. 2023 Sep 22;14:5919. doi: 10.1038/s41467-023-41672-1 (PMC10517010; doi:10.1038/s41467-023-41672-1)
Supplement: Supplementary file 3 — Description of Additional Supplementary Files [file 41467_2023_41672_MOESM3_ESM.pdf]

**Title: Supplementary Data 1**

**Description:** Details of the peptides detected by MS after digestion of acetylated 4R tau.  The digestion of the sample was performed by trypsin enzyme. The acetylation reaction was performed for 12 hours in the presence of both p300 and CBP acetyltransferases.
